# Multisystemic engagement & nephrology based educational intervention: a randomized controlled trial protocol on the kidney team at home-study

**DOI:** 10.1186/1471-2369-13-62

**Published:** 2012-07-23

**Authors:** Sohal Y Ismail, Annemarie E Luchtenburg, Willij C Zuidema, Charlotte Boonstra, Willem Weimar, Emma K Massey, Jan J Busschbach

**Affiliations:** 1 Departments of Medical Psychology and Psychotherapy, Erasmus MC, Burg. s’ Jacobplein 51, Rotterdam, CA, 3015, The Netherlands; 2 Internal Medicine, ‘s Gravendijkwal 230, Rotterdam, CE, 3015, The Netherlands; 3 Department of Medical Psychology and Psychotherapy, Erasmus MC, Postbox 2040, Rotterdam, CA, 3000, The Netherlands

**Keywords:** Family Therapy, Cultural Diversity, Kidney transplantation/psychology, Living Donors/psychology, Patient education, Accessibility of health services

## Abstract

**Background:**

Living donor kidney transplantation (LDKT) is the most successful form of renal replacement therapy in terms of wait time and survival rates. However, we observed a significant inequality in the number of LDKT performed between the Dutch and the non-Dutch patients. The objective of this study is to adapt, implement and test an educational home-based intervention to contribute to the reduction of this inequality. Our aim is to establish this through guided communication together with the social network of the patients in an attempt that well-informed decisions regarding renal replacement therapy can be made: *Multisystemic Engagement & Nephrology*. This manuscript is a detailed description of the Kidney Team At Home-study protocol.

**Methods and design:**

All patients (>18 yrs) that are referred to the pre-transplantation outpatient clinic are eligible to participate in the study. Patients will be randomly assigned to either an experimental or a control group. The control group will continue to receive standard care. The experimental group will receive standard care plus a home-based educational intervention. The intervention consists of two sessions at the patient’s home, an initial session with the patient and a second session for which individuals from their social network are invited to take part. Based on the literature and behavioural change theories we hypothesize that reducing hurdles in knowledge, risk perception, subjective norm, self-efficacy, and communication contribute to well-informed decision making and reducing inequality in accessing LDKT programs. A change in these factors is consequently our primary outcome-measure. Based on power calculations, we aim to include 160 patients over a period of two years.

**Discussion:**

If we are able to show that this home-based group educational intervention contributes to 1) achieving well-informed decision regarding treatment and 2) reducing the inequality in LDKT, the quality of life of patients will be improved while healthcare costs are reduced. As the intervention is investigated in a random heterogeneous patient group in daily practice, the transfer to clinical practice in other kidney transplant centers should be relatively easy.

**Trial registration:**

Netherlands Trial Register, NTR2730.

## Background

Patients with end-stage renal disease have various options for renal replacement therapy (RRT): hemodialysis, peritoneal dialysis, deceased donor kidney transplantation (DDKT) and living donor kidney transplantation (LDKT). Although dialysis is a life-saving treatment, the patient is faced with a substantial loss of quality of life and a significantly increased risk of morbidity and mortality [1]. At least one quarter of the patients die on the wait list for DDKT [2]. The calculation of the time spent on the wait list for a DDKT starts on the first day of dialysis and is on average three to five years in the Netherlands [3]. Research shows that LDKT is associated with significant patient and graft survival benefits when compared to DDKT [4]. One of the benefits of early LDKT is avoiding or minimising time on dialysis. However, one of the main concerns among patients is the health of the living donor. Studies have shown that among healthy screened individuals, the health risks for the donor are limited [5]. The donor is usually admitted for 3–4 days for the nephrectomy, can resume preoperative social and professional activities within four to six weeks and in general the perceived quality of life remains the same [5,6]. LDKT rates have steadily been increasing and now exceed those of DDKT in the Netherlands [3]. However, there appears to be an inequality in access to the LDKT program between Dutch and the non-Dutch patients. In our center 44% of patients on the wait list for DDKT are from non-Dutch descent [7]. However, non-Dutch patients represent only 18% of the patients transplanted via the LDKT program (period: 2000–2010). Therefore, fewer non-Dutch than Dutch benefit from the advantages of LDKT. This inequality is also present in other western countries [8-10]. This health care inequity needs to be addressed [11,12]. This discrepancy is partly attributable to medical, socio-economic and ethnic factors, which exert an independent significant influence on the chance of receiving a LDKT [7]. Due to an accumulation of unfavourable factors in the non-European population, their chances for a LDKT dropped to only 10% compared to the reference population (69%) [13]. Of the socio-economic hurdles, health insurance is less relevant in the Netherlands due to a health insurance system which is accessible for all Dutch citizens. Other possibly contributing factors to this inequality are potentially modifiable psychosocial (e.g. patient education, cognitions and emotions) and culture-specific factors (social influences, communication attitudes) [14,15]. The Kidney Team At Home-study focuses on addressing those potentially modifiable factors in a home-based educational intervention.

In response to this situation, we developed an educational program based on some of the principles of Multi System Therapy (MST) [16]. MST is an evidence-based therapy, which has been developed for derailed adolescents and families. Such serious pathology is unlikely to be found in the current study population. This means that the intervention applied here will be much less intense than MST in a pure form. The current intervention is an adaptation of the intervention developed by Rodrigue, which was also MST-based and proven to be effective in reducing inequality in patients with end-stage renal disease [17]. The intervention was adapted to the Dutch situation with regard to the culture specific factors and the content of the education appropriate to the Netherlands. We designed our intervention with respect to the MST framework in such a way that we strive for engagement of the patient’s family and social network in the disease process: *Multisystemic Engagement & Nephrology*.

We developed an intervention protocol based on empirical data on psychosocial hurdles to LDKT and influential theories from health psychology that focus on decision-making process and behavioural change. A close fit was found between data-driven hurdles and the following theory: Attitude-Social influence-Efficacy model (ASE-Model) [18]. The ASE-Model is based on the theory of Theory of Planned Behaviour (TPB) of Fishbein and Ajzen [19] and is supplemented by elements from the Social Cognitive Theory (SCT) of Bandura [20]. ASE has a wide scientific acceptance and represents a theoretical framework for explaining behaviour by connecting attitude, social influence, self-efficacy, knowledge, skills (communication), and barriers and resources (risk perception) to intention and behaviour. Firstly, the factor regarding ‘*attitude*’ in this theory is based on 1) the belief that people think that a certain behaviour will have positive or negative consequences and 2) their evaluation of the according consequences. In other words, attitude is a function of how we integrate the information that we have on a subject. Secondly, *social influence* is defined as the approval or disapproval of the pursued behaviour by others within the patient’s social network. Thirdly, *self-efficacy* looks at the extent to which individuals believe in their own abilities in relation to a particular behaviour. These factors influence a person’s *intention* to carry out certain behaviour. Empirical data in this area has shown that factors such as *knowledge, risk perception, attitude, communication, social influence, self-efficacy and intention* reveal good predictive values in the light of living organ donation [21-26].

### Objectives

The objective of the Kidney Team At Home-study is to contribute to the reduction of ethnic inequalities in LDKT health care access. We translated this objective in two concrete research questions. The primary research question is to investigate whether this home-based educational intervention results in improved knowledge and communication as compared to the standard educational care. The intervention should in this matter help the patients and their social network to reach a *well-informed decision* with regard to the most suitable treatment option. This is deliberately set as a primary research question, as a well-informed decision does not necessarily have to lead to LDKT. The secondary research question is to investigate whether this intervention leads to reduced ethnic inequality in the pursuit of LDKT. This is operationalized by looking into distribution of LDKT activities between the experimental and control group. This study protocol provides a detailed description of the Kidney Team At Home-study and the design of the randomized controlled trial (RCT) in line with the CONSORT (consolidated standards of reporting trails) checklist [27,28].

## Methods and design

### Study population

#### Patients

Eligible candidates are end-stage renal patients who have been referred to the pre-transplant clinic and are currently listed on the wait list for a DDKT and who do not have a potential living donor yet. This includes patients (>18 yrs) newly referred for transplant preparation as well as patients who are already listed. Patients who are mentally incapable (e.g. mental deterioration, schizophrenic) or those with a compromised medical condition who are unable to withstand the intervention will not be included.

#### Invitees

Individuals in the social network of the patients are also invited to take part in this study. This practice is in line with the multisystemic approach of MST. For the invitees there are no limits set to ethnicity or the relationship with the patient. The number (≥1) of invitees (>18 yrs) participating will depend on the number that responds to the invitation of the patient.

### Design & procedure

In this prospective RCT all patients will be invited to participate by the educators after the consultation with the transplant nephrologists. During the face-to-face informational consultation the patient will receive written and verbal information on the aims and procedures of the study. Spouses, relatives or friends accompanying the patient to the hospital may be present during this consultation. Our target is to include 80 patients of Dutch origin and 80 patients of non-Dutch origin in the study over a period of two years in order to compare effectiveness of the intervention among these two groups. With respect to the inequality in LDKT, patients of non-Dutch origin will inherently be overrepresented in the study. After informed consent is obtained, patients will be randomized to either the control or the experimental group. The control group will receive standard care. The experimental group will receive standard care plus a home-based educational intervention. In the face of the equity principle, we will provide all the study materials (e.g. brochures, questionnaires) in the six most common foreign languages in the Rotterdam municipality namely, English, Arabic, Turkish, Papiamento, Portuguese and Modern Hindi [29].

#### Control condition

Patients assigned to the control group will receive standard care only. In the standard care all patients visiting our pre-transplantation outpatient clinic receive a consultation with a transplant nephrologist, a transplant coordinator, and a social worker. Additional to this verbal information, the patients receive a variety of written educational material and a DVD regarding various living donation and transplantation programs. All materials are translated in the six afore mentioned foreign languages. They can study this material in the coming four weeks before their second visit to the outpatient clinic during which they have the opportunity to ask additional questions. Additionally, our patients are invited to attend 4–6 times a year informational meetings held in the various regional hospitals. The baseline and post-measurements will be either handed out by the educators during the hospital visit or sent via mail. If necessary the educators may help in completing the questionnaire. The questionnaires are also available in six languages.

#### Experimental condition

The intervention consists of two sessions at the patient’s home. Session One: Firstly, at the beginning of the session the patient completes the baseline measurement. Secondly, the family network of the patients will be depicted on a genogram the educator in order to get familiar with the family structure and to recognize the values of that social system. The observation of the educators during the sessions represents an important source of information about how the present individuals relate to each other. In fact, the educators never rely solely on the individual’s verbal self-description to investigate social relations. Only by observing how the individuals behave with each other can the educators support or reject hypotheses based on self-reports. During this first session the educators watch for non-verbal clues that confirm or contradict what the social system is telling them. As the educators form tentative hypotheses about psychosocial hurdles, they do not offer advice or share their observations to avoid defensiveness. They rather focus on engagement by showing helpful interest while listening to the needs of the individuals and reinforcing the strengths of the respective social system. At the end of the first session the educators will make an inventory of individuals that the patient will invite for the second session. The educators may help in inviting identified individuals. The invitation will be conducted verbally accompanied by a brochure containing the 1) study purpose, 2) education content, and 3) contact information of the educators.

Session Two: The educators organize this session in such a way that they will do ‘what ever it takes’, in line with one of the basic principles of MST, to make this event as patient-tailored as possible. This means that the intervention will usually take place in the evenings and weekends since most friends and family members are working during office hours. The primary goal of this intervention is educational, therefore, it is not necessary that all the invitees are potential donors. The baseline-outcome measurement for the invitees will take place at the start of the second session. In exceptional cases multiple sessions are required in order to reach the goals set for those sessions. Table 1 shows the topics that will be discussed during the second session.

**Table 1:** The educational topics discussed in the second session

		
	1 Introduction	The purpose of the Kidney Team At Home-study
	2 Kidney disease	An introduction to kidneys and kidney diseases
	3 Dialysis	The various forms of dialysis
	4	Morbidity and mortality associated with dialysis
	5	The psychosocial consequences of a kidney disease and dialysis
	6	The advantages and disadvantages of dialysis compared to kidney transplantation
	7 Transplantation	The medical evaluation in preparation for donor nephrectomy and kidney transplantation
	8	The various programs of donation and transplantation (DDKT and LDKT)
	9	The number of DDKT and LDKT performed nationally and locally
	10	The differences in ethnicity regarding access to LDKT
	11	The differences in graft survival between DDKT and LDKT
	12 LDKT	Additional advantages and disadvantages of LDKT
	13	The risks and psychosocial aspects associated with donor nephrectomy
	14	The personal, emotional and financial aspects of LDKT for the recipient
	15 Discussions	Whether present individuals have considered donation of their kidney

During both sessions at the patients’ home we will use the therapeutic framework of MST in order to stimulate open communication between the patient and the family members and to use and profit of the strengths and possibilities of the natural network of the patient. The objective of MST is to achieve a lasting consensus on the patient’s goals and how these goals can be reached with engagement and/or support of his/her social ecology. Such long-term consensus can not be achieved if the interpersonal relations, personal autonomy and feelings of those involved are not sufficiently considered. Therefore, creating a ‘safe’ environment during the intervention is regarded as an important aspect of a successfully implemented intervention. The licensed psychologist who will be implementing this protocol is certified in practical systemic therapy. Both the psychologist and the transplant coordinator (educators) will be supervised by an official MST supervisor throughout the study period.

**Figure 1:**
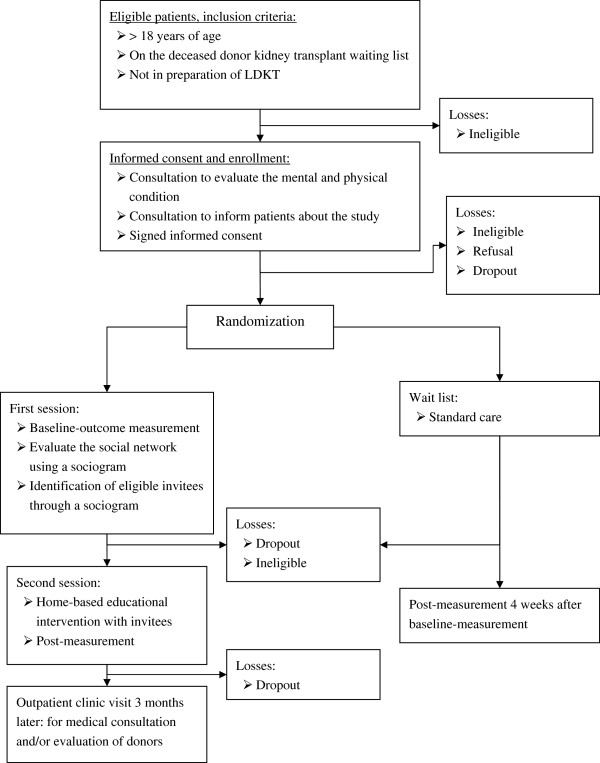
Design of the randomized controlled trial.

In order to minimize hurdles for participation interpreters are used when Dutch is not the primary language of those present. The interpreter will also help patients and invitees with questionnaires or understanding the informed consent if that may ease the transfer of information. At the end of the second session the patient and the invitees will receive the post-measurement. This questionnaire can be completed immediately or returned via the mail within a week. In Figure 1 one can find the graphical depiction of the RCT.

#### Primary outcome measures

##### Knowledge

Because there was no suitable instrument to measure knowledge on kidney diseases and all the possible RRT’s, we developed the Rotterdam Renal Knowledge-Test (RRK-T) based on Devin et al (1990), Stothers et al (2005) and Rodrigue et al (2007). The RRK-T consists on 30 true / false or multiple choice questions on kidney disease, dialysis and transplantation with a living or deceased donor [29]. For example, *‘Renal replacement therapy is necessary when the kidneys function for only 50%’.* A clinically significant change will be indicated by 8 points of difference compared to the baseline measurement and is additional to passing the clinical cut-off point of 17 points (each correctly answered questionnaire is awarded 1 point) [30].

##### Attitude

This is operationalized as the attitude that one has towards the discussion of RRT’s (for patients and invitees) and the acceptance (patients) or the donation (invitees) of a kidney (TPB) [26,31-33]. For example, *‘From my point of view discussing renal replacement therapy with my family and friends is pleasant / unpleasant’ and ‘I think that accepting a kidney from a living donor is good / bad.’* These were rated on a scale from 1–7.

##### Risk perception

The questionnaire structure is similar to other studies in which the PMT is used [34,35]. Negative and positive aspects of living kidney donation were rated on a 5-point scale (1 = not at all - 5 = a lot). For example, *‘I think discussing living kidney donation is emotionally burdensome’* and *‘I am afraid that in the future I could get a kidney disease from kidney donation.’*

##### Communication

Patients and invitees are asked if and how often they talk about kidney diseases and RRT with their family and friends. The development and selection of these questions was based on the validated ‘Family Functioning Questionnaire’ [36]. This questionnaire is designed to pinpoint the way families communicate and make choices regarding psycho-educational interventions. For example, ‘*How often have you recently talked with the people close to you about kidney transplantation from a living donor’ (1 = never - 5 = very often).*

##### Self-efficacy

We will measure the patients’ and invitees self-efficacy to communicate about LDKT. Participants could answer on a 5-point scale ranging from ‘I am able to … …’ to ‘I am not able to …’ [25,37,38]. For example, *‘I am able to discuss renal replacement therapies with my family and friends.’*

##### Subjective norm

Questions will be asked to determine if and how much patients and invitees value the thoughts of the other party regarding LDKT [37]. For example, *‘I value the opinion of people who are important to me’ (1 = not at all- 5 = very much).*

##### Intention

This factor measures the extent to which patients and invitees plan to / are willing to discuss RRT’s or if they would give or accept a kidney [25,26]. For example: *‘How much do you want to donate a kidney to the patient’ (1 = not at all - 5 = very much).*

#### Secondary outcome measures

Secondary outcomes are operationalized in terms of behaviour that may lead to LDKT or LDKT itself. This is divided into three categories: the number of applications for LDKT evaluation, the number of actual evaluations for LDKT, and the number of LDKT’s. These outcomes will be monitored using the patient’s medical records during the year following the intervention date. Furthermore, we will also monitor the timeframe between the intervention and the moment that the event arises (time-to-event).

#### Other outcome variables

Several other factors (e.g. confounders, effect modifiers) that may have an effect on the outcome of the intervention are defined as follows: Satisfaction with the intervention and / the process leading to the well-informed decision for both the patient and the invitees, the number of invitees, the relationship of the invitee to the patient, the duration of the meeting, the use of an interpreter, the number of visits necessary for the intervention, the treatment adherence of the professionals and the satisfaction of the professionals. These outcomes are recorded by the educators on a questionnaire for each intervention.

##### Product evaluation

Patients and invitees will be asked four questions on their opinion of the intervention provided by the educators: satisfaction regarding the educational intervention (e.g. usefulness, clarity). For example, *‘How satisfied are you with the clarity of the received information ’ (1 = very dissatisfied - 5 = very satisfied).*

##### Process evaluation

Patients and invitees will be asked eight questions on their opinion and satisfaction regarding the way in which the intervention is delivered. (e.g. logistics, cooperation, understanding, professionalism). The questions asked are based upon the concepts and the structure of the Revised Treatment Adherence Measure (TAM; [39,40]. The main underlying question of the TAM is: ‘Did the health care providers do what they were ought to do in congruency with their protocol?’ For example, *‘The researchers encouraged communication between me and my family/friends’ (1 = not at all - 5 = to a large degree).*

##### Educator process evaluation

This questionnaire (12 items) will be completed by the co-educator to determine whether the intervention was able to reach the right audience (reach) and whether it was implemented as intended (fidelity) [41]. For example, *‘How satisfied are you with the communicative aspects of the home visit?’ (1 = very dissatisfied - 10 = very satisfied).* Followed by, *‘What went well and what could have been improved?’ (open questions).*

#### Background variables

Socio-demographic data: date of birth, gender, education, employment status, marital status, number of children, ethnicity and religion will be collected through medical records. Medical data: medical diagnosis, history of other RRT’s, current treatment, date of first dialysis and blood type.

#### Sample size calculation

An *alpha* of .05 and a *power* of .80 was used in the following calculations, as proposed to be appropriate for behavioural research [42]. To determine an adequate sample size for detecting the effect we did a power analysis based on the proportion of LDKT’s performed in the control versus the experimental group in previous research [17]. We used this parameter since this is the only one on which there has been reported in the literature with regard to the current study parameters. Moreover the other parameters would reveal inconsistent sample estimates. For example, the knowledge parameter would show a large effect size which would result in a very low sample size whereas, self-efficacy would require a lager sample size. The required sample sizes to achieve a nominal power of 1-γ = 0.8 on a two-sided test with a α = 0.05 using a Fisher distribution revealed that at least 78 patients are required per study group to enable statistical judgments that are accurate and reliable. Calculations were performed in SAS; Power and Sample Size version 3.1.

#### Statistical analysis

Following the updated CONSORT statement [43], for this study the intention-to-treat population is defined as all randomized patients who are known to have received at least one home visit and who provide data for at least one post-baseline measurement for one or more of the key efficacy variables: no patient will be excluded for protocol violations which occurred during subsequent follow-up (modified intention-to-treat) [44]. Additional effort will be exerted by the educators to ask the drop-out patients to complete the post-measurement for the primary outcome.

For comparing the patients’ baseline-variables between the two research conditions the two tailed t-test for independent samples for the continuous variables, the two-tailed Mann–Whitney U-tests for the ordinal variables and the Chi-square tests for the categorical variables will be used. The effectiveness of our home-based educational intervention for the primary outcome variables will be analyzed with mixed modeling, i.e. multilevel regression modeling. The additional value of this multilevel testing lies in the flexibility to model individual growth trajectories and to handle missing data. The latter will only hold if structure of the missing data is Missing Completely At Random (MCAR) or Missing At Random (MAR), but not if the data turns out to be Missing Not At Random (MNAR). This technique is appropriate since missing data and drop-outs are inevitable in longitudinal research. Moreover, missing data affects study power simply by the reduced availability of data points [45]. We will strive to firstly minimize missing data, and to subsequently take this into account during analysis. The methodology of mixed modeling for repeated measures allows us the use of flexible error variance-covariance structure. Additionally, the predictive value of the baseline-parameters (main-outcome variables) on effectiveness can be estimated.

Finally, semiparametric regression analysis will be employed using Cox Proportional Hazard Model to examine the significance of the contingency between the hazard for an event of the experimental and control group on the secondary outcome variables. This model enriches the analysis by incorporating covariates in the regression equation (e.g. age, gender, ethnicity, dialysis time). Time-to-event graphs will be depicted for the experimental and control group separately. Patients with a LDKT will be regarded as having the ‘event’ whereas patients who continue dialysis during the follow-up period will be censored. DDKT is modeled as a competing event and therefore patients who take-up this treatment will also be censored.

### Ethical considerations

The Medical Ethical Committee of Erasmus Medical Center, Rotterdam, The Netherlands, has approved this study, registered under MEC-2011-004 / NL34535.078.10. Firstly, the ethical feasibility of an intervention as we propose in this protocol had been evaluated. In that research it had been argued that an active intervention in peoples’ live is justified [46]. The proposed education and therapeutic counseling in this protocol needs to be relative to the social context and the personal condition of the subsequent patients in order to ensure ethical justification. Secondly, in our center we have recently published an article on the ethical considerations of such a home-based educational intervention for kidney patients and their social network [47]. The authors concluded that a home-based approach is ethically justified when certain essential conditions had been satisfied. We consider the following as the most important: 1) participation must be completely voluntary at any point during the intervention, 2) the intervention must not be persuasive (i.e. advocating for a certain treatment option), and 3) the goal and the procedure of the intervention must be clear to all participants.

## Discussion

LDKT has become a successful commonplace treatment option. The donor is often a family member but can also be a friend or an acquaintance in the Netherlands [48]. This has led to a broadened range of potential living donors. The graft survival rates of LDKT are better than those of the deceased donor transplantation [49]. Although research and clinical experience have shown LDKT to be a better alternative for patients with end-stage renal disease, the uptake of LDKT remains stagnant in culturally diverse populations. This inequity needs to be addressed [12,14,47]. Consequently, our aim was to address this inequality within a therapeutic framework that has already been scientifically established. We integrated the principles of MST within health behavioural change theories to create this home-based educational intervention. Combining these principles with the results of previous research on psychosocial and culture specific potentially modifiable hurdles [14,15] has resulted in the Kidney Team At Home study. The primary research question of this study is to investigate whether a home-based educational intervention is effective for end-stage renal patients in reducing hurdles to LDKT. The secondary research question is to investigate whether the intervention increases the rates of LDKT among ethically diverse populations. We will try to establish this through guided communication, which if effective, can support and encourage well-informed decision making. Discussing a difficult to broach topic such as living donation, which hitherto could not be adequately discussed [51], may be an emotional burden to the patients and their social network. Within the MST framework it is the explicitly framing of this very burden that is crucial to the intervention. With this multisystemic approach one seeks to resolve existing tensions in collaboration with the participants and try to pave the way for the emergence of a stable and suitable consensus on the issue at hand. This consensus will embrace a well-informed decision, which can be the pursuit of LDKT but may also be (continuation of) dialysis. The discussion of living donation is for health care professionals not one of the toughest conversations one can imagine. Indeed, the discussion should be seen as a complicated but nevertheless as a mild social dilemma for the educators. Since MST is an evidence-based therapy, we believe that MST is able to provide us with tools that allow us to safely frame the transfer of information and the discussion of LDKT in the family network of the patient.

### Other ongoing studies

In our center we are currently also investigating a multicenter home-based intervention in a cross-over design [47]. However, the target population is different for that study compared to the KTAH-study. The target population are pre-emptive patients, who are Dutch speaking, and without a history of other renal replacement therapies. Additionally, in contrast to the KTAH-study, this pre-emptive study focuses on primary knowledge provision without implementing a psychotherapeutically intervention. Another culturally sensitive study protocol has been recently published [52]. However, also that study focuses on pre-emptive patients.

### Practical considerations

Kidney patients in the experimental group are hosts for the sessions at their home. In most cases the patient and his/her family appreciate this informal setting and the fact that they do not have to make extra visits the hospital to participate in this study. However, if the patient does not wish to have the session at their home it may be held at a different location (e.g. community center, church). An additional flexibility in this protocol is that we will provide them with interpreters who were trained on the matter at hand for those to whom the Dutch language may be a barrier. Finally, if the per protocol number of visits appears not to be sufficient enough to reach the pre-specified goal by the educators, multiple sessions may be held in agreement with the participants. All these flexibilities are justified under the ‘what ever it takes’ principle of MST and is being done to make the intervention easily accessible for the patients and the individuals from their social network.

## Conclusion

If we show that this method is effective in reducing hurdles for LDKT the hypotheses will hold that patients will reach more stable and well-informed decision regarding renal replacement treatment options together with their social network. This supplementary home-based educational intervention may contribute to reducing inequalities in access to LDKT by addressing specific psychosocial and cultural hurdles at a grass-roots level.

## Competing interest

The authors declare that they have no competing interest.

## Authors’ contributions

JJVB, WW, EKM and WCZ developed the original idea of the study, submitted a grand application to the Dutch Kidney Foundation, and all supervised the implementation of the project. SYI and AEL wrote the study protocol, the ad verbatim *Multisystemic Engagement & Nephrology* manual and are the educators who are implementing the study in the outpatient pre-transplant clinic of the Erasmus Medical Center, Rotterdam, The Netherlands. CB, contributed to the development of the protocol, our therapeutic multisystemic framework and will continue to supervise the implementation throughout. All authors read and corrected the draft versions. All authors approved the final version of this protocol.

## Pre-publication history

The pre-publication history for this paper can be accessed here:

http://www.biomedcentral.com/1471-2369/13/62/prepub
